# 

**Published:** 1895-08

**Authors:** 


### CONTENTS.

ORIGINAL ARTICLES:

An Outbreak of Epizoötic Abortion in Cattle. By Veterinary Surgeon DESMOND . . . 469

A Suggestion or Aid to the Eradication of Tuberculosis by a Practical and Profitable Method. By A. S. HEATH, M.D., V.S. . . . 474

A New Cause of Death of Cattle. By N. S. MAYO, D.V.S. . . . 477

Stable Hygiene. By J. W. SALLADE, V.S. . . . 480

Actinomycosis. By W. C. BARTH V.S. . . . 487

A Selection in Cattle as a Preventive of Disease Among People. By JOHN ERNST, V.S. . . . 490

Reminiscences of Castration. By M. J. TREACY, M.R.C.V.S. . . . 492

Some Every-day Points in Veterinary Jurisprudence. By JOSEPH N. B. RAWLE, L.B., M.D.S., V.S. . . . 496

Address. By S. STEWART, M.D., D.V.M. . . . 497

REPORTS OF CASES:

Paralysis of the Nerve Radialis. By OTTO NOACK, V.S. . . . 501

Tumors of the Brain Disclosed by Autopsy. By H. D. GILL, V.S. . . . 502

CORRESPONDENCE:

Tetanus . . . 503

Inspection of Dairies. By J. H. WATTLES, V.S. . . . 509

LEGISLATION—New York—Massachusetts . . . 512–515

AMONG THE COLLEGES:

Harvard Veterinary Department—Veterinary Department, University of Pennsylvania—Veterinary Department, McGill University—New York College of Veterinary Surgeons . . . 516–517

EDITORIALS:

Notice to Our Readers—Convention of College Faculties—What is the Matter with American Veterinary Journalism?—The New Outlook—Our Friend Bryden 518–520

SELECTIONS:

An Appeal for the Selection of a Proper Meat and Milk Inspector. By OTTO NOACK, V.S. . . . 521

PERSONAL—U. S. V. M. A.—NECROLOGY—REVIEW—WHAT THE WORLD IS DOING—SOCIETY PROCEEDINGS—NEW INVENTIONS—POSITION TO EXCHANGE 524–535

